# Abietane Diterpernoids from the Roots of *Euphorbia ebracteolata*

**DOI:** 10.1007/s13659-018-0159-9

**Published:** 2018-03-20

**Authors:** Yuan-Liang Ma, Xiao-Han Tang, Wen-Juan Yuan, Xiao Ding, Ying-Tong Di, Xiao-Jiang Hao

**Affiliations:** 1 State Key Laboratory of Phytochemistry and Plant Resources in West China, Kunming Institute of Botany, Chinese Academy of Sciences, Kunming, 650201 People’s Republic of China; 2 University of Chinese Academy of Sciences, Beijing, 100049 People’s Republic of China; 3 Yunnan Institution for Food And Drug Control, Kunming, 650011 People’s Republic of China

**Keywords:** Euphorbiaceae, *Euphorbia ebracteolata*, Abietane diterpernoid, Cytotoxic activity

## Abstract

**Abstract:**

A new *ent*-abietane diterpernoid, named ebracteolata D (**1**), along with 11 known analogues, was isolated from the roots of *Euphorbia ebracteolata* Hayata. The structure of **1** was elucidated on the basis of spectroscopic analysis and molecular modeling. Cytotoxicity of compounds **1**–**12** was evaluated as well as the effect on the NF-κB pathway. Among them, compound **12**, jolkinolide B, displayed broad inhibitory effects against proliferation of tumor cell lines. Mechanistic studies indicated that the compound **12** can inhibit TNF-α induced NF-κB activation, thereby inducing tumor cell apoptosis.

**Graphical Abstract:**

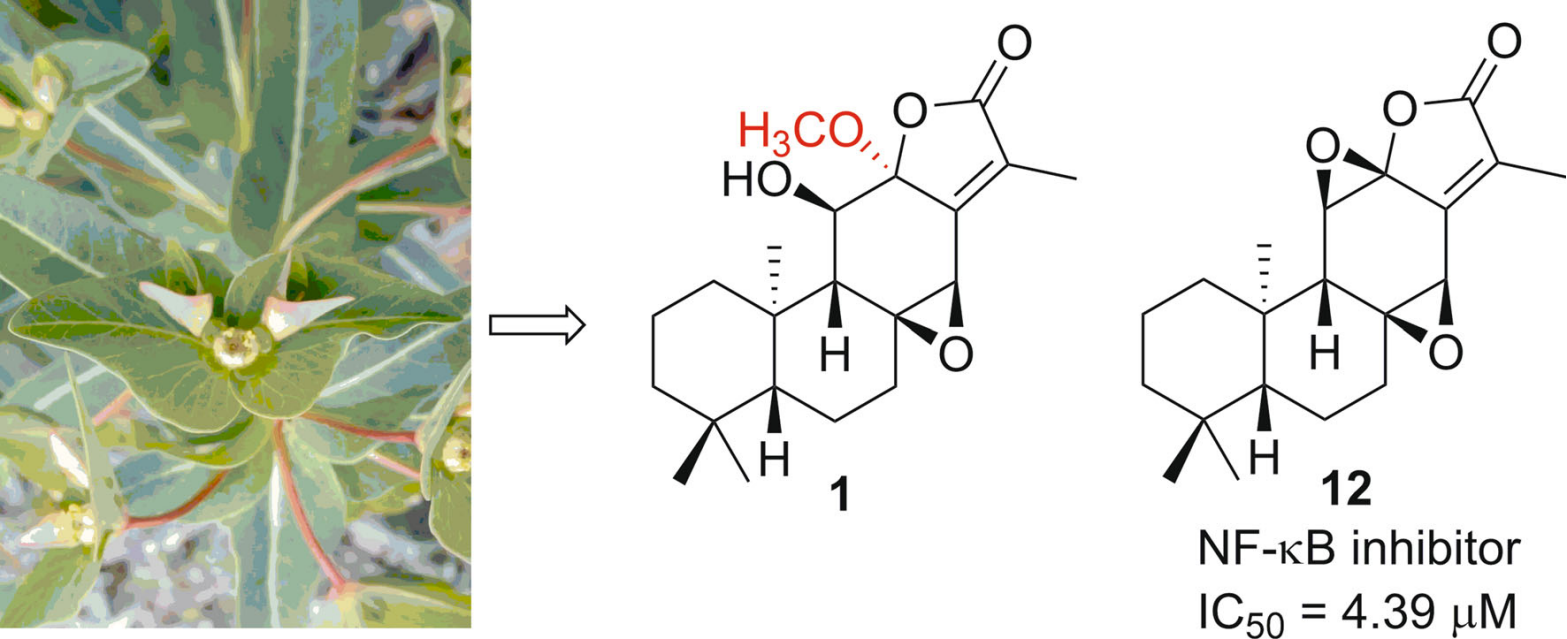

**Electronic supplementary material:**

The online version of this article (10.1007/s13659-018-0159-9) contains supplementary material, which is available to authorized users.

## Introduction

NF-κB is a key transcription factor playing important role in tumor progression and drug resistance [1–3]. Suppression of NF-κB activation could enhance the efficacy of anticancer drugs and overcome drug resistance [4]. Several NF-κB inhibitors have been employed as sensitizers in established cancer therapy over recent years. For example, a combination of NF-κB inhibitor, such as parthenolide or curcumin, with PXL has been demonstrated to augment the therapeutic efficacy in various cancer models [5].

**Fig. 1:**
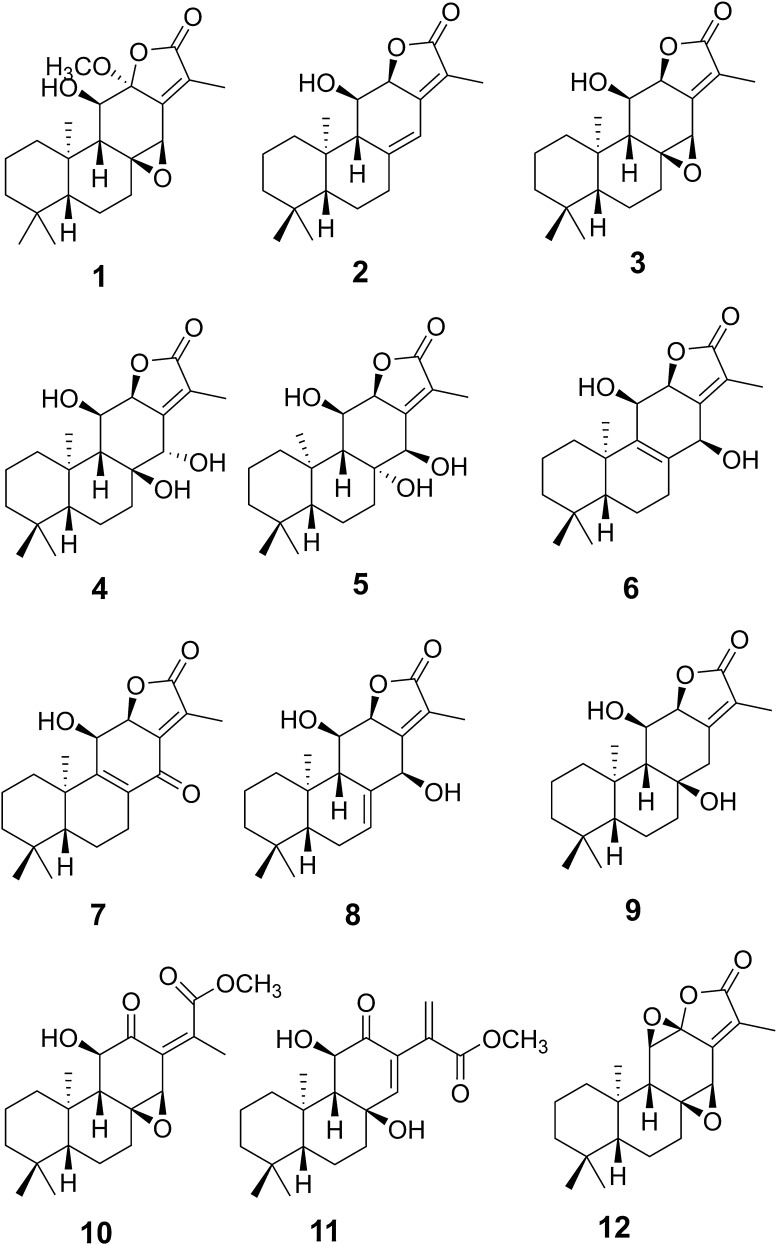
The structures of compounds **1**–**12**

Euphorbiaceae is one of the largest families of higher plants, comprising about 500 genera, 5000 species, and widely distributes in the world, especially in the tropical and subtropical regions [6]. The genus *Euphorbia* is the largest in the spurge family, some of which have been used as medicinal plants for a long time [7, 8]. Among them, the roots of *Euphorbia ebracteolata* are used to treat pulmonary tuberculosis, chronic tracheitis, and psoriasis in Traditional Chinese Medicine (TCM) [9]. Till now, a number of diterpenoids with a wide spectrum of bioactivities, including antihepatotoxic and cytotoxic activities, have been isolated from this species [10–12]. In order to identify additional biologically active diterpenoids, a chemical investigation of the roots of *E. ebracteolata* was performed [13]. As a result, one new *ent*-abietane diterpernoid, ebracteolata D (**1**), together with 11 known analogues was obtained from petroleum ether-soluble extracts. The known compounds were identified as 11*β*-hydroxy-*ent*-abieta-8(14),13(15)-dien-16,12*α*-olide (**2**) [14], ebracteolatanolide A (**3**) [15], yuexiandajisu D (**4**) [16], yuexiandajisu E (**5**) [16], ebractenoid K (**6**) [17], ebractenoid L (**7**) [17], ebractenoid M (**8**) [17], tetrahydrojolkinolide B (**9**) [18], euphorin H (**10**) [19], methyl-8*β*,11*β*-dihydroxy-12-oxo-*ent*-abieta-13,15(17)-dine-16-oate (**11**) [20] and jolkinolide B (**12**) [14] by comparison of their spectroscopic data with those reported in the literature. Herein, we report the isolation, structure characterization, and bioactivity evaluation of these compounds (Fig. 1).

## Results and Discussion

The 95% EtOH extract of the roots of *E. ebracteolata* Hayata was fractionated between EtOAc and H_2_O. The EtOAc fraction was subjected to repeated chromatography, to yield 12 *ent*-abietane dierpernoids (**1**–**12**).

Ebracteolata D (**1)** was obtained as a white powder. The molecular formula was determined as C_21_H_30_O_5_ on the basis of HRESIMS (*m/z* 385.1993 [M + Na]^+^), indicating seven degrees of unsaturation. The IR absorptions at 1757 cm^−1^, in combination with UV absorption maxima at 233, and 308 nm, implied the presence of an α,β-unsaturated-*γ*-lactone moiety in the molecule, which was confirmed by the ^13^C NMR signals (*δ*_C_ 169.8, 151.4, and 130.5). The 1D NMR data also afford four methyls, one methoxyl (*δ*_H_ 3.24, s, *δ*c 51.2). five *sp*^3^ methylenes, four *sp*^3^ oxymethines (*δ*_C_ 73.9/*δ*_H_ 3.69, C-11; *δ*_C_ 57.4/*δ*_H_ 3.77, C-14), and two *sp*^3^ oxygenated quaternary carbons (*δ*_C_ 106.5, 65.2). The spectroscopic data and the known chemotaxonomy of this genus suggest a skeleton of *ent*-abietane diterpernoid (Table 1).

**Table 1:** ^1^H (600 MHz) and ^13^C (150 MHz) NMR spectroscopic data of ebracteolata D (**1**) in CDCl_3_

No.	*δ*_C_, type	*δ*_H_ (*J* in Hz)	No.	*δ*_C_, type	*δ*_H_ (*J* in Hz)
1*α*	38.9 CH_2_	1.77^a^ (m)	9	59.0 CH	1.81 (d, 3.4)
1*β*		1.25 (m)	10	39.5 C	
2*α*	18.2 CH_2_	1.49 (m)	11	73.9 CH	3.69 (dd, 9.0, 3.4)
2*β*		1.43 (m)	12	106.5 C	
3*α*	41.5 CH_2_	1.41 (m)	13	151.4 C	
3*β*		1.18 (m)	14	57.4 CH	3.77 (s)
4	33.3 C		15	130.5 C	
5	53.2 CH	1.05 (dd, 12.5, 2.1)	16	169.8 C	
6*α*	20.8 CH_2_	1.77^a^ (m)	17	8.8 CH_3_	2.03 (s)
6*β*		1.45 (m)	18	33.5 CH_3_	0.92 (s)
7*α*	34.8 CH_2_	2.05 (m)	19	22.0 CH_3_	0.84 (s)
7*β*		1.58 (m)	20	14.8 CH_3_	0.67 (s)
8	65.2 C		OCH_3_	51.2 CH_3_	3.24 (s)
11-OH		2.97 (d, 9.0)			

^a^Overlapped

Except for the *α*, *β*-unsaturated-*γ*-lactone moiety, the presence of additional four rings was necessary to meet the required number of unsaturation degrees. Since three rings of an *ent*-abietane diterpernoid accounted for three degrees of unsaturation, there should be another ring. One oxymethine at *δ*_H_ 3.77 and *δ*_C_ 57.4, along with an oxygenated quaternary carbon at *δ*_C_ 65.2, suggested the presence of an epoxy ring between C-8 and C-14. This was supported by the HMBC correlations from H_2_-7 (*δ*_H_ 1.58, 2.07) to C-8 and C-14, and from H-14 (*δ*_H_ 3.77) to C-8.

Complete assignments of proton and carbon signals were performed by 2D NMR experiments. According to ^1^H-^1^H COSY and HSQC spectrum, the following protonated partial structures were established: C-1/C-2/C-3, C-5/C-6/C-7, and C-9/C-11. The HMBC correlations readily established A and B rings, which were the same as those of jolkinolide B.^14^ In the HMBC spectrum, cross-peaks of H-11/C-12, and H-14/C-12 and C-13 showed the presence of six-membered ring C, including C-8, 9, 11, 12, 13 and C-14. Moreover, methoxyl at C-12 was determined by the HMBC correlations between OCH_3_ (*δ*_H_ 3.24, *δ*c 51.2) and C-12. The remaining methyl (*δ*_H_ 8.8, *δ*_C_ 2.03) assigned to the C-17 was linked with C-15 as judged from the correlations from Me-17 to C-13, C-15, and C-16. Although no direct HMBC correlation was available to link C-12 and C-16, from the degrees of unsaturation and their chemical shift, the ester bond between both the carbons could be assumed. The planar structure of Ebracteolata D (**1**) was thus elucidated as indicated.

**Fig. 2:**
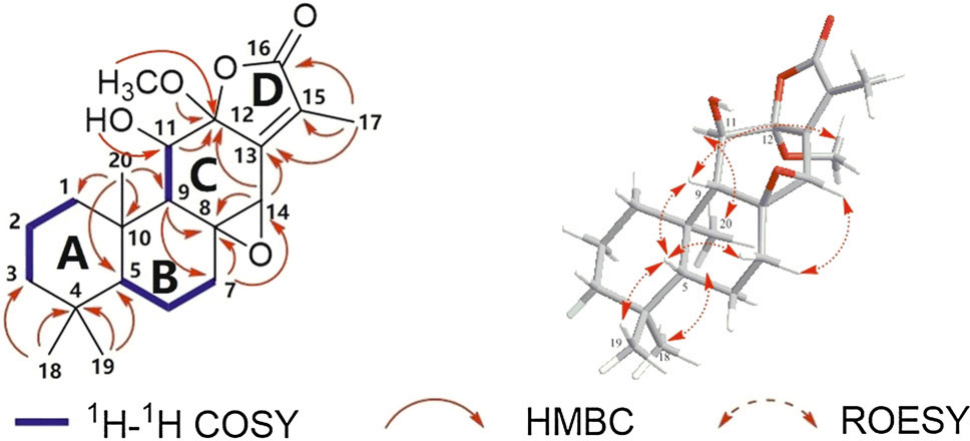
key ^1^H–^1^H COSY ( ), HMBC ( ) and ROESY ( ) correlation for **1**

**Fig. 3:**
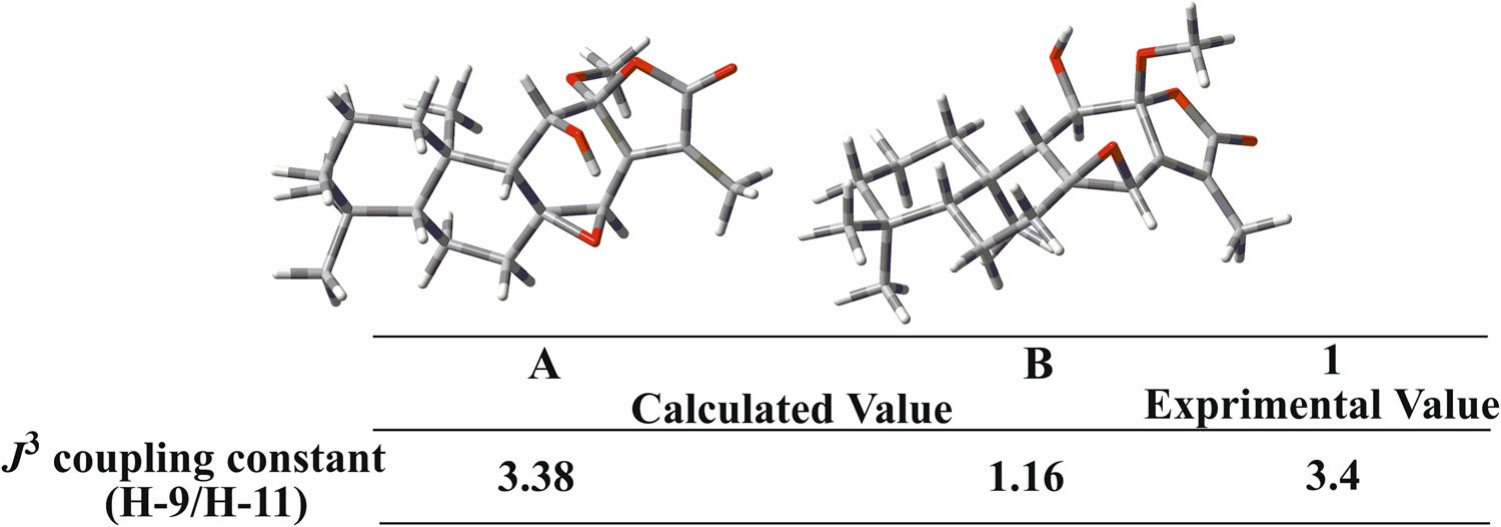
Two possible structures (**A** and **B**) of **1**. Calculated and experimental *J*^3^-coupling constants between H-9 and H-11 are also available

The relative configuration of **1** was fixed by ROESY experiment and molecular modeling (Conflex 7 Rev. C). The orientation of Me-18 was arbitrarily assigned as *α*. In the ROESY spectrum, the cross peaks observed between the proton pairs Me-1/Me-20, H-11/ Me-20 indicated that H-11 and Me-20 were on the same side towards *α*-orientation (Fig. 2). The ROE correlations of Me-19/H-5, H-5/H-9, and H-9/H-7*β* suggested that Me-19, H-5, H-9 and H-7*β* took *β*-configuration. Moreover, the orientation of the C-8/C-14 epoxy ring was determined to be *β* due to the ROE correlation between H-7*α* and H-14, which was identical to that of jolkinolide B. However, due to flexibility of the OCH_3_ in the structure of **1**, it is not sufficient to determine the orientation of the OCH_3_. Therefore, molecular modeling was performed for the two possible structures of **1**, corresponding to the *α* (**A**) or *β* (**B**) orientation of the OCH_3_ as shown in Fig. 3. Two optimized structures were obtained, in which the calculated *J*^3^-coupling constant between H-9 and H-11 in **A** was fully consistent with the corresponding NMR data. Therefore, the configuration of OCH_3_-12 was determined to be *α*.

Compounds **1**–**12** were evaluated for their cytotoxicity against five human tumor cell lines (HL-60, SMMC-7721, A549, MCF-7, and SW480) by the MTS method [21–25]. Only compound **12** showed cytotoxicity against these cell lines with the IC_50_ values at 5.2, 3.8, 11.9, 16.2, and 10.2 μM, respectively. The inactivity of **1**–**11** and activity of **12** in the tested cell lines may be due to their structural different, which suggested that both the C-8/C-14 and C-11/C-12 epoxy ring systems may be essential for these types of biological activity. Then, we evaluated the effects on the NF-κB pathway of these compound**s**. Similarly, only compound **12** showed a moderate inhibitory effect on TNF-*α*-induced activiation of NF-κB pathway with an IC_50_ 4.39 μM. All the results implied that jolkinolide B (**12**) exhibited cytotoxicity via suppressing the activation Nf-κB signaling pathway.

## Experimental Section

### General Experimental Procedures

Optical rotations data were measured with SEPA-300 and Jasco DIP-370 polarimeter. ESIMS spectra were acquired on Waters 2695 HPLC-Thermo Finnigan LCQ Advantage and HRESIMS API Qstar Pulsar spectrometer. A Bio-Rad FTS-135 spectrophotometer was used for IR spectra as KBr pellets. UV spectra were obtained using a Shimadzu UV-210A spectrophotometer. ^1^H, ^13^C NMR and 2D NMR spectra were recorded on Bruker AV-400, DRX-500 and AVANCE III-600 MHz spectrometer with TMS as internal standard. Semi-preparative HPLC separations were performed on an Agilent 1100 liquid chromatograph with a Merck i.d. (10 × 100 mm) column. Column chromatography (CC) was performed using silica gel (200–300 mesh and 60–80 mesh, Qingdao Marine Chemical, Inc., Qingdao, P. R. China) and Sephadex LH-20 (40–70 mesh, Amersham Pharmacia Biotech AB, Uppsala, Sweden). Lichroprep RP-18 gel (40–63 μm; Merck, Darmstadt, Germany). And spots were visualized by heating silica gel plates sprayed with 5% H_2_SO_4_ in EtOH.

### Plant Material

The roots of *E. ebracteolata* were collected from Anhui Province, People’s Republic of China, in November 2014. The plant samples were identified by Prof. Ji-Ming Xü of Kunming Institute of Botany, Chinese Academy of Sciences (CAS). A voucher specimen (HXJ20141108) was deposited at the State Key Laboratory of Phytochemistry and Plant Resources in West China, Kunming Institute of Botany, Chinese Academy of Sciences (CAS).

### Extraction and Isolation

The air-dried, powdered plant materials (20 kg) were extracted with 95% EtOH under reflux three times. The combined EtOH extracts were concentrated under vacuum to give a crude residue (2 kg), which was suspended in water and then partitioned with petroleum ether. The petroleum ether portion (780 g) was subjected to passage over a silica gel column, eluted with a gradient of petroleum ether/ethyl acetate (from 100:0 to 0:100), to yield five major fractions (1–5). Fr.2 (60 g) was then chromatographed on a silica gel column eluted with petroleum ether/acetone (25:1), to get **2** (0.6 g), **3** (45 mg) and Fr.2-3. Fr.2-3 was purified by Sephadex LH- 20 (Acetone) and semipreparative HPLC to give **1** (5 mg), **10** (11 mg), **11** (4 mg) and **12** (400 mg). Fr3 (65 g) separated over a C18 silica gel column (MeOH/H_2_O from 2:5 to 4:5) to obtain four further fractions. Fr.3-2 (9 g) was purified by Sephadex LH-20 column (Acetone) to give **6** (20 mg) and **7** (30 mg). Fr3-3 was chromatographed on a silica gel column eluted with petroleum ether/acetone (15:1), and semipreparative HPLC to get **8** (19 mg) and **9** (16 mg). Fr.4 (50 g) was then separated over a C18 silica gel column (MeOH/H_2_O from 2:5 to 4:5) to obtain four fractions, then Fr.4-1 chromatographed on a silica gel column eluted with petroleum ether/acetone (5:1) and Sephadex LH-20 (Acetone) column to obtain **4** (10 mg) and **5** (28 mg).

### Cytotoxic Activity Assay

The following human tumor cell lines were used: HL-60, SMMC-7721, A-549, MCF-7, and SW-480. All cells were cultured in RPMI-1640 or DMEM medium (Hyclone, Logan, UT, USA), supplemented with 10% fetal bovine serum (Hyclone) at 37°C in a humidified atmosphere with 5% CO_2_. Cell viability was assessed by conducting colorimetric measurements of the amount of insoluble formazan formed in living cells based on the reduction of 3- (4,5-dimethylthiazol-2-yl)-5(3-carboxymethoxyphenyl)-2-(4-sulfopheny)-2*H*-tetrazolium (MTS) (Sigma, St. Louis, MO, USA). Briefly, 100 mL of adherent cells was seeded into each well of a 96-well cell culture plate and allowed to adhere for 12 h before test compound addition, while suspended cells were seeded just before test compound addition, both with an initial density of 1 × 105 cells/mL in 100 mL of medium. Each cell line was exposed to the test compound at various concentrations in triplicate for 48 h, with cisplatin and paclitaxel (Sigma) used as positive controls. After the incubation, MTS (100 mg) was added to each well, and the incubation continued for 4 h at 37 °C. The cells were lysed with 100 mL of 20% SDS-50% DMF after removal of 100 mL of medium. The optical density of the lysate was measured at 595 nm in a 96- well microtiter plate reader (Bio-Rad 680). The IC_50_ values of each compound were calculated by Reed and Muench’s method. Activity evaluation methods such as literature method, cisplatin was used as positive control (cisplatin showed cytotoxicity the IC_50_ values at 18.80 (HL-60), 12.32 (A-549), 17.50 (SMMC-7721), 20.50 (MCF-7), 12.01 (SW-480) μM, respectively).

### NF-κB Luciferase Reporter Assay

NF-κB pathway activity was tested by the dual luciferase reporter gene assay method. EK 293T cells were seeded in 24-well plates and transiently transfected with 5 × κB-luciferase and pTK-Renilla reporters using Lipofectamine 2000 for 18 h. Cells were then incubated with different concentrations of compounds for different times, and subsequently stimulated with 10 ng/mL TNF-α for 4 h. Luciferase activity of cell lysate was analyzed using the Dual Luciferase Reporter Assay System (Promega).

## Electronic supplementary material

Below is the link to the electronic supplementary material.

Supplementary material 1 (PDF 1167 kb)
