# Supplementary material for: Abietane Diterpernoids from the Roots of Euphorbia ebracteolata
Source: Nat Prod Bioprospect. 2018 Mar 20;8(2):131–5. doi: 10.1007/s13659-018-0159-9 (PMC5913052; doi:10.1007/s13659-018-0159-9)
Supplement: Supplementary file 1 — Supplementary material 1 (PDF 1167 kb) [file 13659_2018_159_MOESM1_ESM.pdf]

Supplementary Data

Abietane Diterpernoids from the roots of *Euphorbia ebracteolata*

Yuan-Liang Ma ·Xiao-Han Tang ·Wen-Juan Yuan · Xiao Ding · Ying-Tong Di · Xiao-Jiang Hao

Y-L Ma · X-H Tan · X Ding · W-J Yuan · Y-T Di (✉) · X-J Hao (✉)

State Key Laboratory of Phytochemistry and Plant Resources in West China, Kunming Institute of Botany, Chinese Academy of Sciences, Kunming 650201, China.

email: diyt@mail.kib.ac.cn, haoxj@mail.kib.ac.cn

Y-L Ma

University of Chinese Academy of Sciences, Beijing, 100049, China.

W-J Yuan

Yunnan Institution for Food And Drug Control, Kunming 650011, China

Table of Contents

No.	Contents	Page
1	**Figure S1** ^{1}H NMR spectrum of Ebracteolata D (**1**) in $CDCl_3$-d_1	2
2	**Figure S2** ^{13}C NMR spectrum of Ebracteolata D (**1**) in $CDCl_3$-d_1	2
3	**Figure S3** COSY spectrum of Ebracteolata D (**1**) in $CDCl_3$-d_1	3
4	**Figure S4** HSQC spectrum of Ebracteolata D (**1**) in $CDCl_3$-d_1	3
5	**Figure S5** HMBC spectrum of Ebracteolata D (**1**) in $CDCl_3$-d_1	4
6	**Figure S6** ROESY spectrum of Ebracteolata D (**1**) in $CDCl_3$-d_1	4
7	**Figure S7** HRESIMS spectrums of Ebracteolata D (**1**)	5
8	**Figure S8** UV spectrum of Ebracteolata D (**1**) in Methanol	6
9	**Figure S9** IR spectrum of Ebracteolata D (**1**)	7
10	**Table 1** The effects of compound **12** on Nf-κB activation with or without TNF-*α*.	8

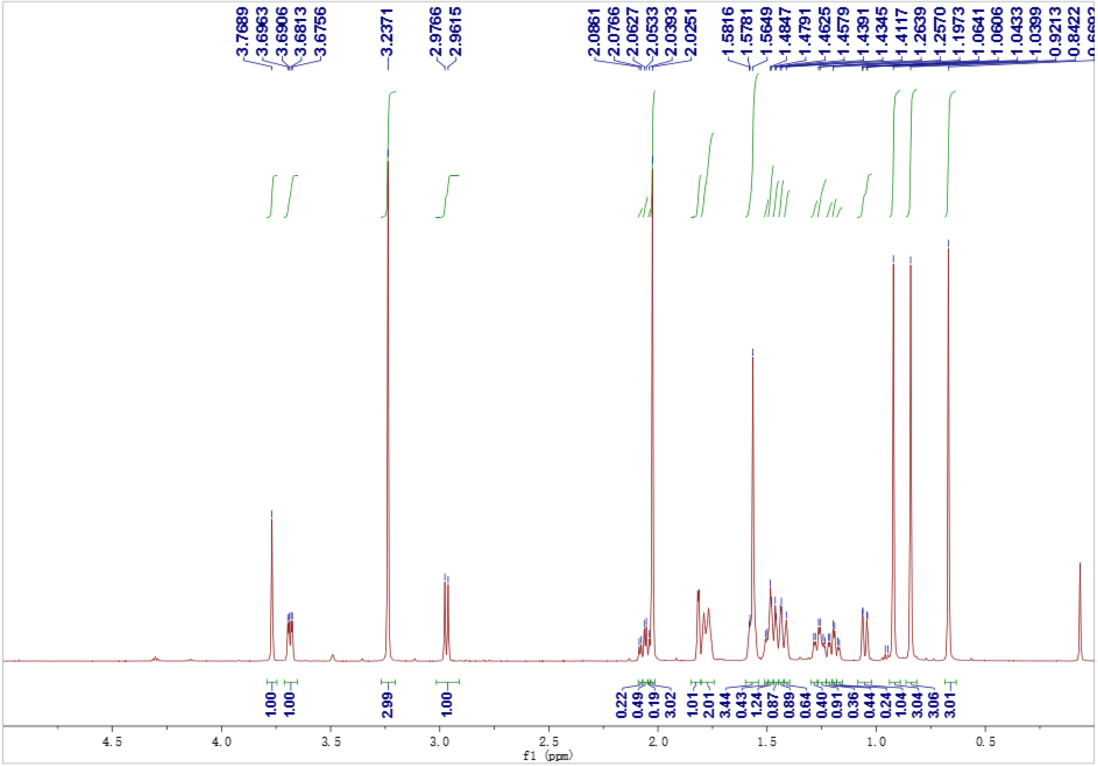

S1 ^{1}H NMR spectrum of Ebracteolata D (1) in $CDCl_3$-*d*$_1$

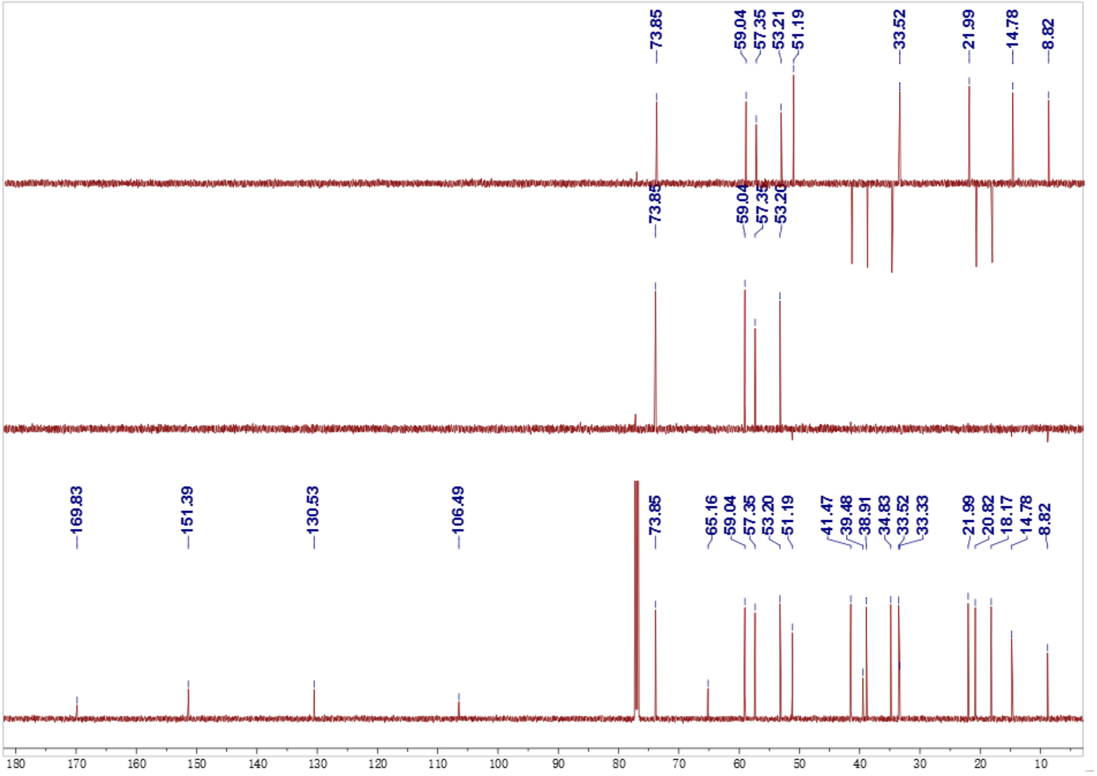

S2 ^{13}C NMR spectrum of Ebracteolata D (1) in $CDCl_3$-*d*$_1$

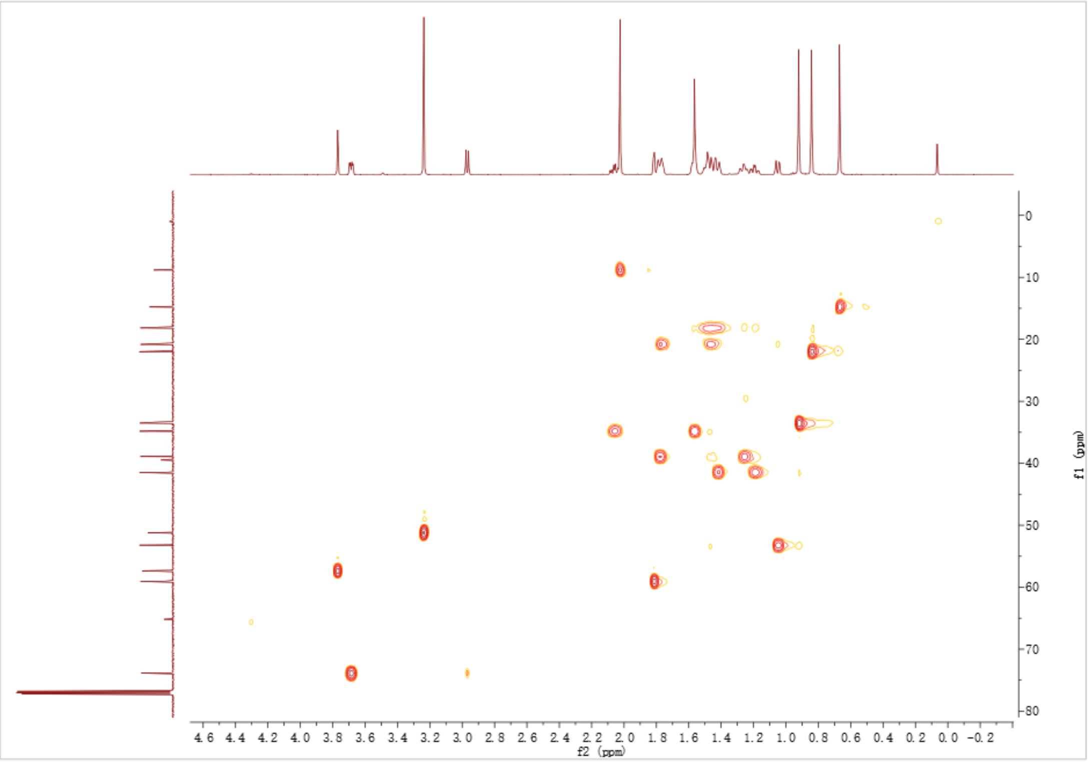

S3 HSQC spectrum of Ebracteolata D (1) in $CDCl_3$-*d_1*

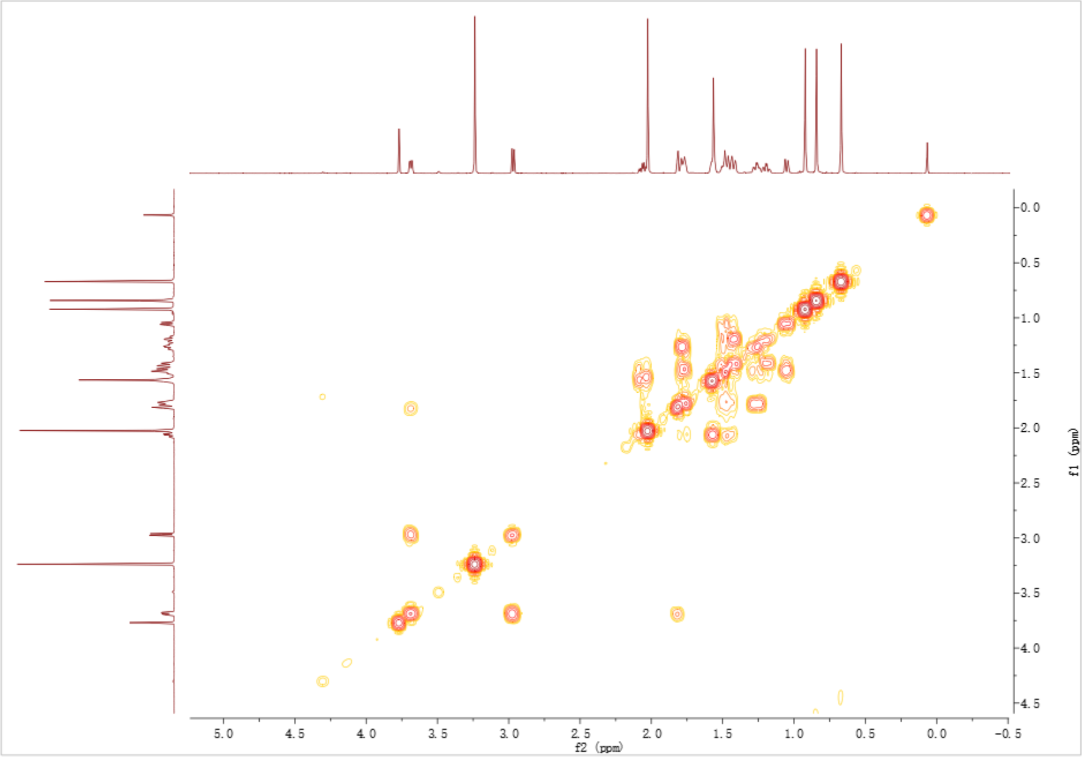

S4 COSY spectrum of Ebracteolata D (1) in $CDCl_3$-*d_1*

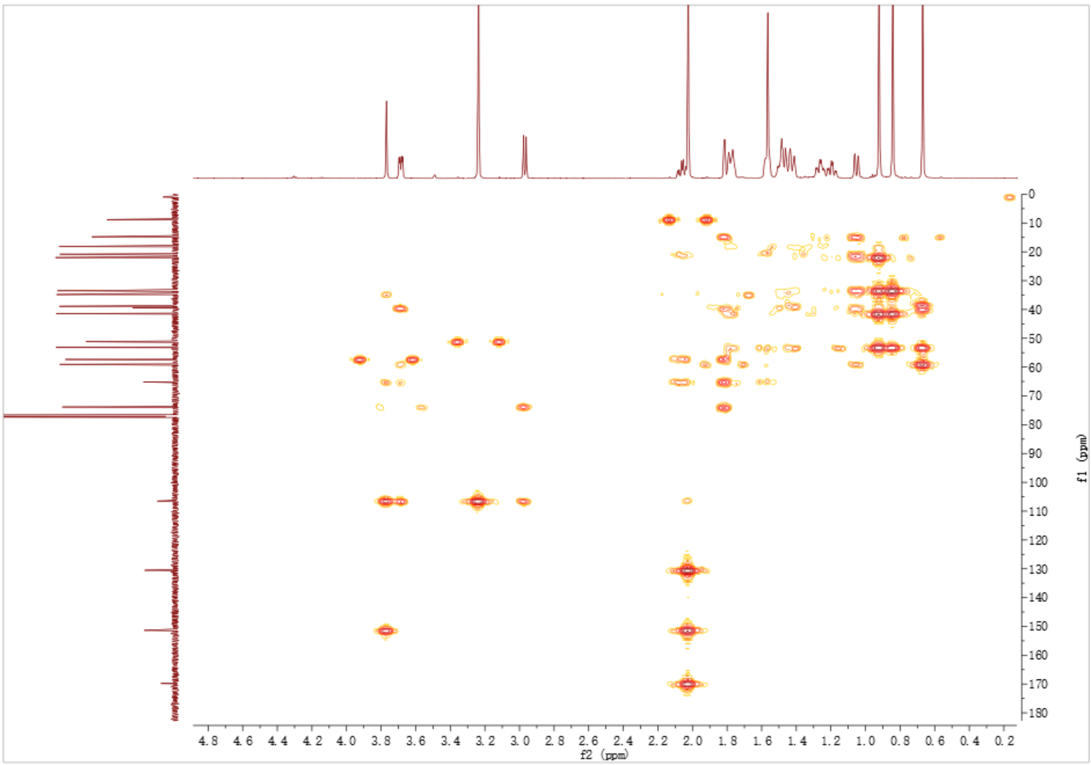

S5 HMBC spectrum of Ebracteolata D (1) in CDCl3-*d*1

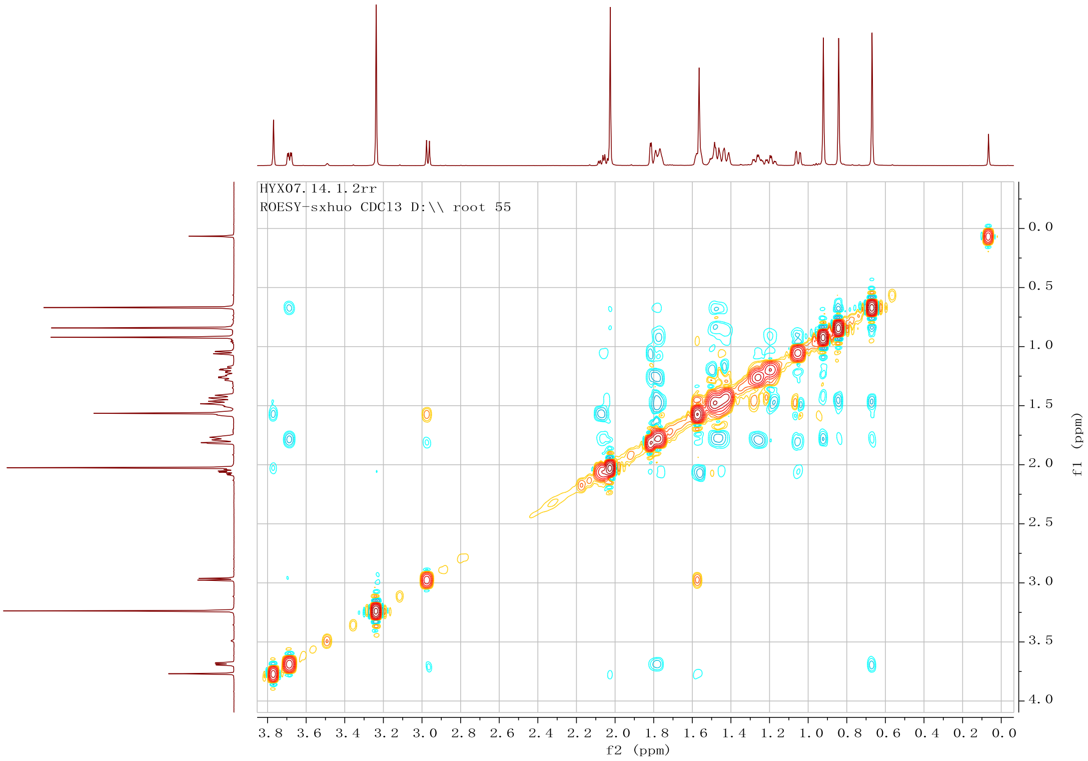

S6 ROESY spectrum of Ebracteolata D (1) in CDCl3-*d*1

Qualitative Analysis Report

**Data Filename**	150515ESIA6.d	**Sample Name**	hyx7
**Sample Type**	Sample	**Position**	
**Instrument Name**	Agilent G6230 TOF MS	**User Name**	KIB
**Acq Method**	ESI.m	**Acquired Time**	5/15/2015 10:11:54 AM
**IRM Calibration Status**	Success	**DA Method**	ESI.m
**Comment**			

Sample Group

Info.

Acquisition SW Version 6200 series TOF/6500 series Q-TOF B.05.01 (B5125.2)

User Spectra

Fragmentor Voltage	Collision Energy	Ionization Mode
200	0	ESI

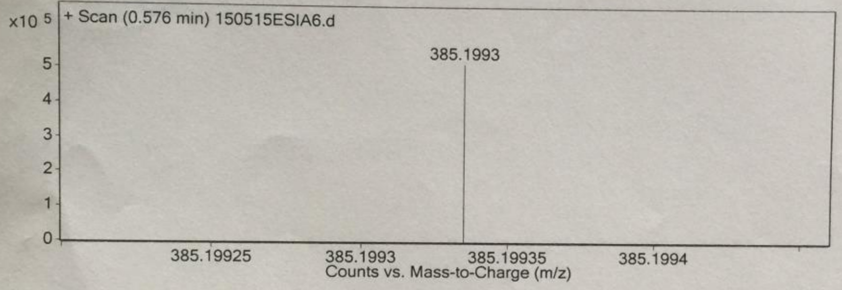

Peak List

m/z	z	Abund	Formula	Ion
274.2742	1	204810.08		
318.3005	1	166792.38		
384.3087	1	150627.7		
385.1993	1	516021.94	C21 H30 Na O5	M+
386.2022	1	104222.04	C21 H30 Na O5	M+
747.408	1	1667909.88		
748.4126	1	772401.81		
749.4153	1	197110.06		
1109.6179	1	398226.41		
1110.6212	1	267778.75		

Formula Calculator Element Limits

Element	Min	Max
C	0	200
H	0	400
O	0	10
Na	1	1

Formula Calculator Results

Formula	CalculatedMass	CalculatedMz	Mz	Diff. (mDa)	Diff. (ppm)	DBE
C21 H30 Na O5	385.1991	385.1985	385.1993	-0.7	-1.8	6.5000

--- End Of Report ---

Agilent Technologies Page 1 of 1 Printed at: 10:20 AM on: 5/15/2015

S7 HRESIMS spectrums of Ebracteolata D (1)

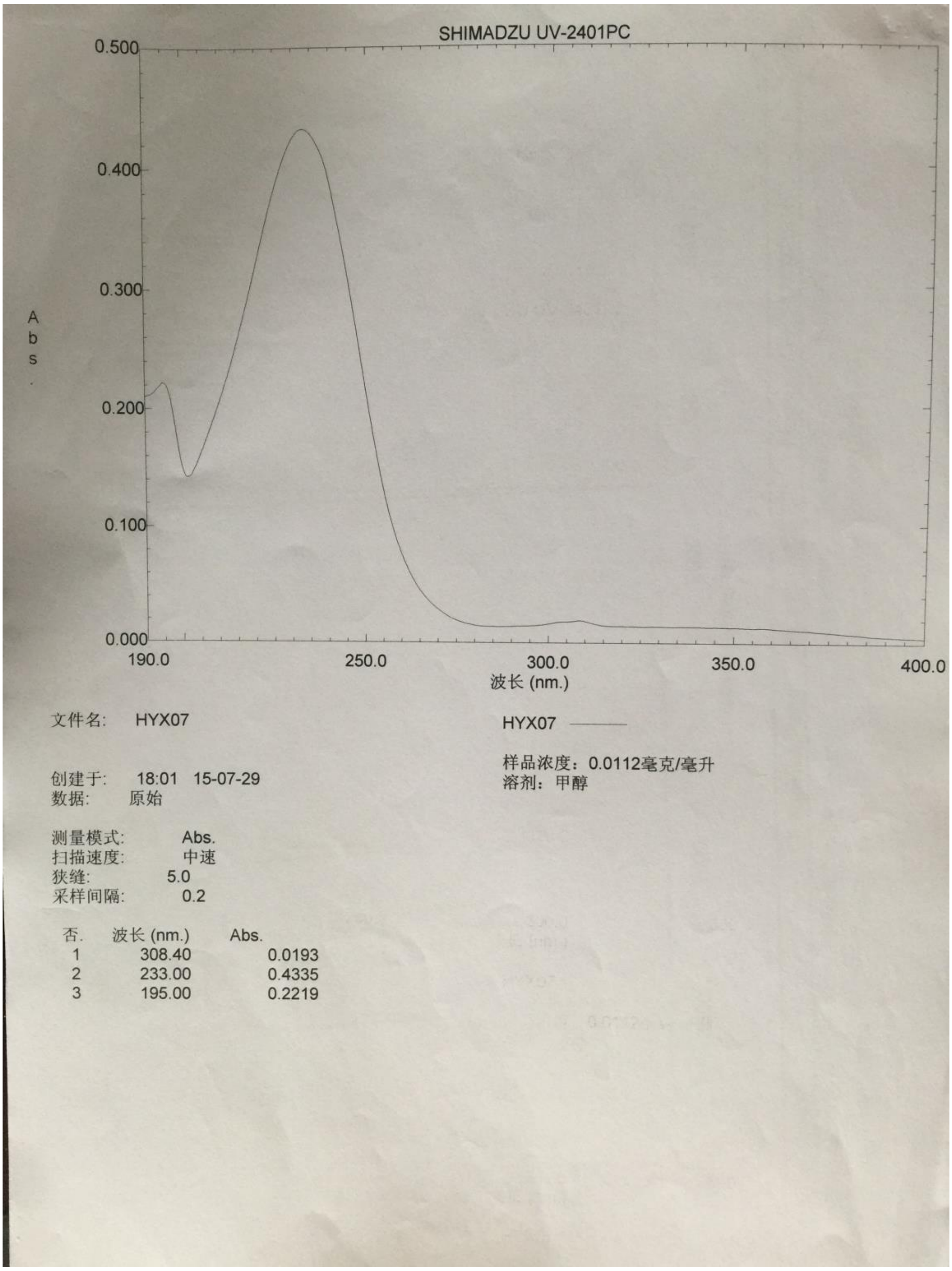

文件名: HYX07

HYX07 ———

样品浓度：0.0112毫克/毫升
溶剂：甲醇

创建于: 18:01 15-07-29
数据: 原始

测量模式: Abs.
扫描速度: 中速
狭缝: 5.0
采样间隔: 0.2

否.	波长 (nm.)	Abs.
1	308.40	0.0193
2	233.00	0.4335
3	195.00	0.2219

S8 UV spectrum of Ebracteolata D (1) in Methanol

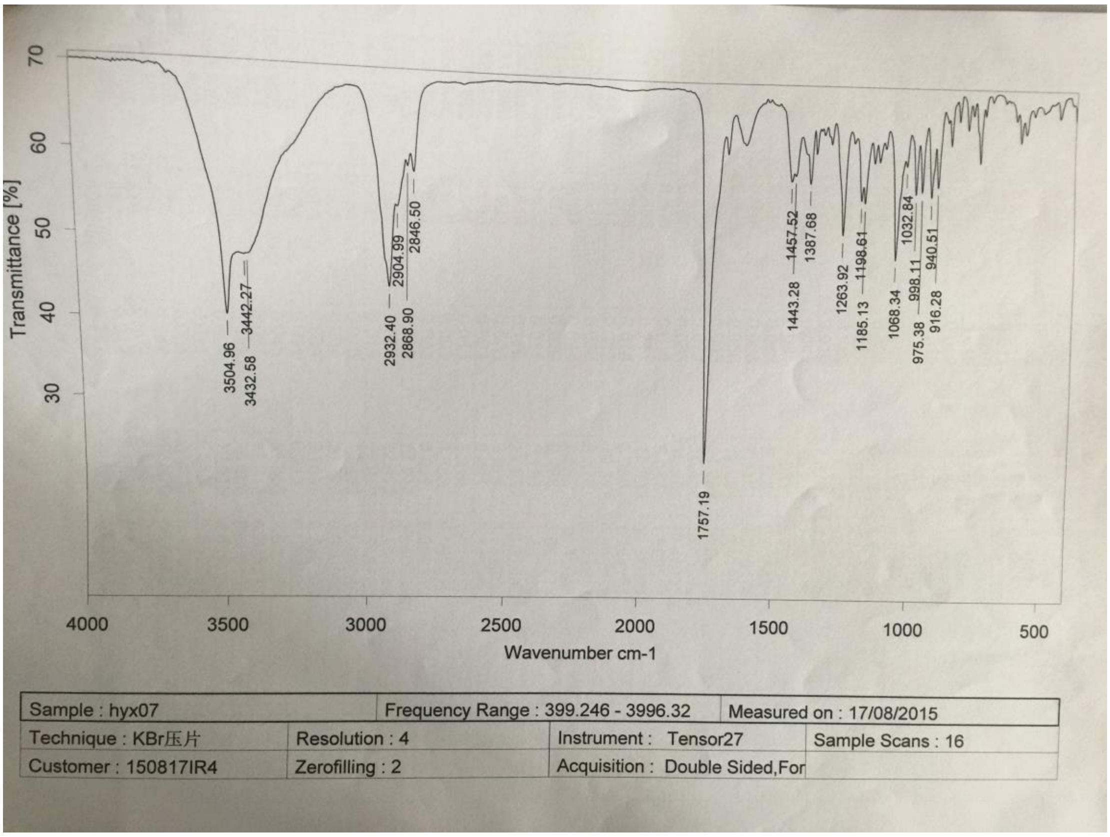

S9 IR spectrum of Ebracteolata D (1)

Table 1 The effects of compound 12 on Nf-κB activation with or without TNF-*α*.

		实验结果（n=3）		
blank		0.075	0.026	0.434
ctrl（TNF-*α*）		12.299	12.193	13.332
**12**	20 *μ*m（TNF-*α*）	0.722	0.575	0.507
	10 *μ*m（TNF-*α*）	2.007	1.897	2.114
	5 *μ*m（TNF-*α*）	7.485	4.733	5.003
	2.5 *μ*m（TNF-*α*）	8.602	9.004	8.685
